# Post-Fall Care in Older Adult Psychiatric Inpatient Settings: Identifying Variations and Implementing Standardised Medical Review Practices

**DOI:** 10.1192/bjo.2026.11400

**Published:** 2026-06-30

**Authors:** Ruzaika Jaufer, Marcelle McDonald-Leslie, Anto Varughese, Hugo Ben Scholfield

**Affiliations:** 1 Norfolk and Suffolk NHS Foundation Trust, Norwich, United Kingdom; 2 Norfolk and Norwich University Hospital NHS Foundation Trust, Norwich, United Kingdom

## Abstract

**Aims:**

This quality improvement project aimed to strengthen the timeliness, consistency and quality of post-fall medical assessments and documentation across older adult inpatient wards. National standards, including NICE Quality Standard 86 and Royal College of Physicians’ recommendations, emphasise prompt review and comprehensive assessment following inpatient falls. Local incident reviews, however, indicated delays in medical examination, inconsistent documentation, and variation in practice. The project aimed to evaluate current practice, identify gaps, and inform the development of an improved post-fall medical review template, as well as contribute to the revision of the Trust’s Falls Policy.

**Methods:**

A retrospective review of 30 inpatient fall incidents was conducted across six older adult mental health wards at the Julian Hospital and Carlton Court Hospital over a three-month period. Data were extracted from incident reports and clinical records, focusing on:

• Whether a post-fall medical review was completed.

• Time from fall to medical assessment.

• Completeness and quality of documentation.

• Differences between witnessed and unwitnessed falls.

• Variation by time of day.

Performance was compared against NICE QS86 (statements 4, 5, 6), Trust Falls Policy C86, and national audit benchmarks.

**Results:**

Medical reviews were not consistently completed, with approximately 40% of falls lacking documented medical assessments. Among documented reviews, median time toassessment was 8.7 hours, within the 12-hour standard but indicating avoidable delay, especially for unwitnessed falls and out-of-hours incidents. Documentation quality varied significantly, with omissions in neurological examination, pain assessment, medication review, and rationale for monitoring. Reviews conducted during evening/night shifts tended to be shorter and less comprehensive. Unwitnessed falls showed higher rates of missing neurological observations and inconsistent application of Trust guidance. Overall, substantial variation existed across wards.

**Conclusion:**

The current post-fall medical assessment practice showed significant gaps in both timeliness and documentation quality, creating potential risks for undetected injuries. This highlighted the need for a more structured and standardised approach across older adult psychiatric wards.

Based on the findings of this QI project, a new, user-informed post--fall medical review template was developed to support clearer, more comprehensive documentation. Alongside this, a wider rewrite of the Trust’s Slips, Trips and Falls Policy (C86) is underway. This ongoing work aims to make the policy more detailed, practical, and clinically informative, ensuring staff have clearer guidance when managing both witnessed and unwitnessed falls.

Next steps include staff engagement, implementation of the revised tools, and a re-audit after three months to measure improvement in timeliness, consistency, and quality of post-fall medical care.

